# A plug-and-play microfluidic device for hydrogel fiber spinning†

**DOI:** 10.1039/d4lc00783b

**Published:** 2025-02-06

**Authors:** Kongchang Wei, Wuchao Wang, Giorgia Giovannini, Khushdeep Sharma, René M. Rossi, Luciano F. Boesel

**Affiliations:** a Empa, Swiss Federal Laboratories for Materials Science and Technology, Laboratory for Biomimetic Membranes and Textiles Lerchenfeldstrasse 5 9014 St. Gallen Switzerland; b Empa, Swiss Federal Laboratories for Materials Science and Technology, Laboratory for Biointerfaces Lerchenfeldstrasse 5 9014 St. Gallen Switzerland

## Abstract

Hydrogel fibers are promising biomaterials for a broad range of biomedical applications, including biosensing, drug delivery, and tissue engineering. Different types of microfluidic devices have been developed for hydrogel fiber spinning, however, they often require skillful fabrication procedures with special instruments such as 3D printers and clean-room facilities. On the other hand, microfluidic devices with predetermined and fixed configurations are susceptible to clotting, contamination, and damage, thereby creating a significant barrier for potential users. Herein, we describe a plug-and-play (PnP) microfluidic device for hydrogel fiber spinning. The PnP device was designed to be assembled in a modular manner based on simple mounting of PDMS elastomers on commercial Lego® blocks. Easy disassembly and re-assembly make the device user-friendly, since cleaning or replacing individual modules is convenient. We demonstrated the application of our PnP microfluidic device in alginate (Alg) hydrogel fiber spinning by using a single-module or double-module device. Moreover, thanks to the PnP approach, multi-layered fibers can be produced by using a triple-module device. As proof-of-principle, we fabricated pH-sensitive multi-layered fibers that could be used for monitoring biological environments, showcasing the potential of such a PnP device in advancing biomedical research related to functional fibers.

## Abstract 2

A Lego-inspired microfluidic chip was developed for plug-and-play applications in the production of hydrogel fiber biomaterials.

## Graphical abstract

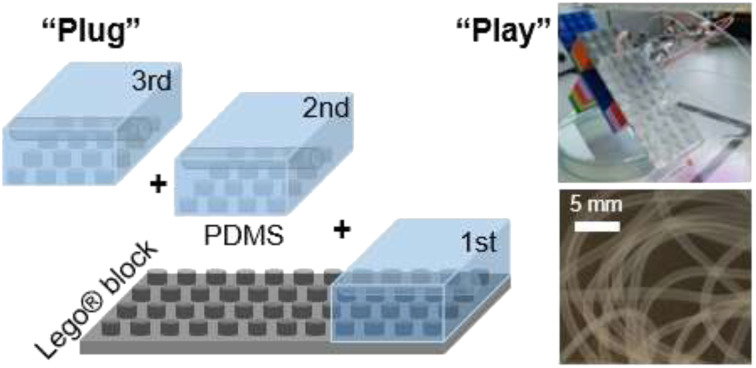

## 1. Introduction

Hydrogel fibers are emerging biomaterials with a unique combination of the high-water-content characteristics of hydrogels and the morphological feature of fiber materials.^1^ Their potential in many biomedical applications, such as drug delivery^2,3^ tissue engineering^3–5^ and biomedical optics^6^ has been intensively explored in the past decade, thanks to the advancement of microfluidic wet spinning technologies for controlled hydrogel fiber fabrication.^1^

The production of fiber materials *via* microfluidic wet spinning requires to shape polymer precursor flows hydrodynamically, therefore the process was also termed “hydrodynamic spinning”.^7^ It relies much on the design and development of reliable microfluidic devices. Such devices with embedded micro-channels are indispensable for controlling the flow of polymer precursor solutions prior to fiber formation.^8^ For this purpose, various types of microfluidic devices have been reported. Among them, polydimethylsiloxane (PDMS)-based microfluidic devices represent the vast majority, due to the unique properties of PDMS, including excellent biocompatibility, mechanical stability, and optical transparency.^9^ PDMS has various commercially available forms, with Sylgard 184® from Dow Corning as the most widely used one. The commercial availability, together with the quick and easy thermal curing procedure, has made Sylgard 184® PDMS suitable for production of cost-effective microfluidic platforms. Micro- or nano-channels can be developed in PDMS by using soft lithography techniques,^9^ which can achieve complex channel designs, however, requires special skills and instruments, such as clean room facilities. Alternatively, channels with relatively simple configurations can be developed by molding, which represents a much more feasible method that can be used by the vast majority of research labs.^10^ Molded channels within PDMS can be used alone for flow control^10^ or integrated easily with glass nozzles and capillaries for more complex configurations.^11^

Recent advance in hydrodynamic spinning has involved other types of microfluidic devices. For instance, glass capillary-based devices combining cylindrical and rectangular tubes were assembled into co-axial channel configurations for hydrogel fiber processing.^12,13^ Stainless steel needles were inserted into a 3D-printed device for the development of triple-orifice spinneret.^7^ Rapid prototyping of microfluidic devices has also been demonstrated by using solely 3D printing technologies.^14–16^ Previously, the combination of soft PDMS elastomer and rigid glass nozzles has been used for co-axial microfluidic devices with fixed configurations.^11,17,18^ In addition to the excellent transparency and biocompatibility, the glass-sealing capacity of PDMS soft elastomer makes such PDMS–glass (soft–hard) hybrid device an attractive option for biomedical microfluidics.^9^ On the one hand, co-axial channel configurations can be facilely established by the combination of PDMS channels and glass nozzles. This is not trivial for conventional PDMS-only microfluidic devices. On the other hand, compared to those fabricated solely from glass capillaries, PDMS–glass hybrid devices can avoid the usage of additional materials (*e.g.*, glues and plastic connectors),^12^ meanwhile reducing the device rigidity.

Nevertheless, existing microfluidic devices for hydrodynamic spinning of fibers have usually fixed channel configurations. The problems of unwanted damage, clotting, and contamination make them difficult to be re-used. For many would-be users from the field of hydrogel fiber biomaterials, a microfluidic spinning system will be of great interest if it can be developed without special instruments and used in a modular “plug-and-play” (PnP) way.

Modular microfluidic devices are those that can be built by connecting multiple microfluidic components together, thereafter the resultant larger integrated system can be disassembled and re-configured.^19^ This approach involves prefabrication of individual microfluidic assembly blocks that can be readily assembled to form microfluidic devices. Modular microfluidic devices were initially developed for biochemical analysis,^20–22^ microreactors,^23^ and laminar flow control.^24,25^ Advanced modular microfluidic systems were also developed for detection of bacterial pathogens.^26^ as well as for microfabrication of polymeric nanoparticles^27^ or droplets.^28,29^ In particular, a few modular microfluidic systems inspired by the world-known Lego® design have been demonstrated,^19,30^ and used for different applications, including organ-on-chip,^31^ stent degradation test and cell culture.^32^ However, modular microfluidic devices for hydrodynamic spinning of fiber materials are yet to be developed.

**Fig. 1:**
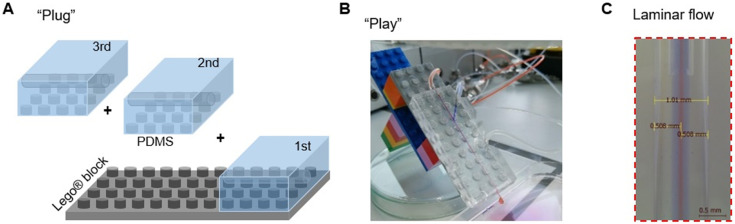
A “plug-and-play” microfluidic device for hydrogel fiber spinning. (A) Schematic illustration of the “plug” step for device assembly. (B) A digital photo of an assembled microfluidic device ready for “play”. (C) A digital photo of the laminar flow established by the PnP microfluidic device.

Not like microfluidic fabrication of nano- or micro-droplets/capsules, microfluidic wet spinning of micro-fibers requires the in-channel continuous solidification of a large amount of polymer mass, which could very often encounter unwanted device clotting and channel contamination induced by the flow disturbance. The difficulty in de-clotting, cleaning, adjusting and re-assembling of fixed-configuration devices is challenging for new users, and even familiar users working with new materials systems. Herein, a Lego®-inspired PDMS–glass hybrid platform was designed (Fig. 1), which enables the facile fabrication, modular assembly, and easy disassembly/re-assembly of microfluidic devices for fiber spinning. With this modular platform, hydrodynamic spinning of alginate (Alg) hydrogel fibers was demonstrated with a single-module device. With the double-module device, a more complex configuration with double-coaxial feature was achieved for spinning hydrogel micro-tubes. Moreover, it was demonstrated that, by expanding the system to a triple-module configuration, multimaterial hydrogel fibers with core–shell–sheath tri-layered structures and pH sensitivity can be spun. These results thus show the feasibility and promise of such a PnP microfluidic platform in developing hydrogel fiber-based materials.

## 2. Materials and methods

Sodium alginate (W201502-1KG), calcium chloride, polyethylene glycol (PEG900kD), deacetylated chitosan, fluorescein isothiocyanate and cold water fish gelatin were purchased from Sigma-Aldrich and used as received. Sylgard® 184 kit (Dow Corning) was purchased from Suter-Kunststoffe AG, Fraubrunnen, Switzerland. Phosphate-buffered saline (PBS) (10 mM) was prepared by solubilizing NaCl (0.137 M), KCl (2.7 mM), Na_2_PO_4_ (0.01 M) and KH_2_PO_4_ (1.8 mM) in DI water. The pH was adjusted by adding NaOH and HCl. Standard Lego® parts were purchased from local shops. Glass capillaries and nozzles made from borosilicate 3.3 were purchased from Hilgenberg GmbH (Germany), with customized dimensions. Glass capillaries products (outer diameter OD = 1 ± 0.025 mm; inner diameter ID = 0.722 ± 0.05 mm) with original length (*L*) as 150 ± 0.5 mm can be customized in the lab by cutting. For glass nozzles, they are customized with total *L* as 40 ± 1.0 mm, OD of the tube as 1 ± 0.025 mm, ID of the tube as 0.722 ± 0.05 mm; *L* of the tip as 20 ± 1 mm, OD of the tip as 0.6 ± 0.1 mm, ID of the tip as 0.4 ± 0.1 mm.

### 2.1 Design of the Lego®-based mold

The modular microfluidic platform was inspired by Lego® products. With a Lego® plate as a base, numerous different modules can be assembled and aligned together to form an integrated design. In order to produce PDMS blocks with a built-in core channel and Lego® features on the bottom, the mold was designed as illustrated in Fig. S1.† Specifically, it was prepared by assembling Lego® parts (standard size with stud feature *D* = 4.8 mm, *H* = 1.7 mm) and a glass capillary (*L* = 11 cm, OD = 1 mm, ID = 0.722 mm). The glass capillary was fixed by double-sided tape at the bottom of the holes in two Lego® bricks.

### 2.2 Molding of PDMS blocks with core channels and Lego® features

The assembled mold was then flipped over and placed in a glass petri dish (Fig. S2A†). The PDMS pre-polymer and the cross-linker (Sylgard® 184, Dow Corning) were mixed in a 10 : 1 ratio, poured into the dish, and then allowed to be degassed under vacuum for bubble removal at room temperature. The PDMS was cured at 80 °C in an oven for 2 hours before demolding. To facilitate the demolding, the glass dish with cured PDMS was immersed in an ethanol bath for 30 min. With the lubrication provided by ethanol, the Lego® blocks and glass capillary were carefully removed from the PDMS elastomer (Fig. S2B†). The molded PDMS elastomer with a built-in core channel and negative Lego® stud feature was then cut into individual modules with designed dimensions (Fig. S2C†). The side inlets were created by direct coring with a cone needle tip (18G). The side inlets created with such a coring method have smaller channel diameters than the 18G needle outer diameter, thus assuring the tight connection with unmodified 18G needles for non-leaking fluid in-flowing.^33^

### 2.3 Assembly of PDMS–glass microfluidic modules and chips

**Fig. 2:**
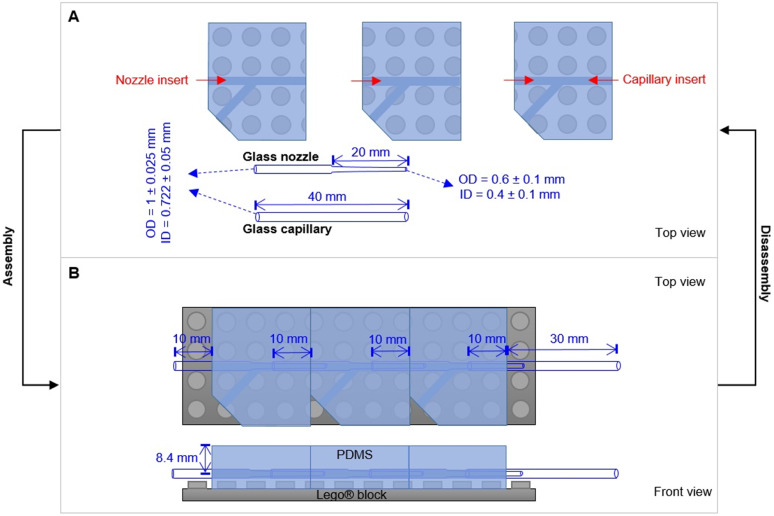
Design principle of the PnP microfluidic device for easy assembly and disassembly. (A) Individual parts of the device. (B) An assembled device with three modules.

Microfluidic chips with different modules were assembled with the individual parts. Firstly, a glass nozzle were inserted into each PDMS block with ethanol as lubricant (Fig. 2). 10 mm of the tube was left outside the PDMS block, either for connection with the liquid supply (1st module), or for connection with the neighbour module (*e.g.*, 2nd and 3rd module). For the last module (*e.g.* 3rd module in Fig. 2B), a glass capillary was inserted from the other direction, with 10 mm in the PDMS block. Secondly, the PDMS–glass individual blocks were connected to each other by the glass nozzles. The complete system was placed on a Lego® plate. The alignment of core channels from different modules were ensured by the multi-point stud features. Such multi-point mounting assembly also locked the neighboring modules together, which were bridged by the tubular section of the glass nozzles for preventing leakage. Disassembly of such a chip was achieved by unplugging the PDMS blocks from the Lego® plate, and dis-connecting the individual blocks from each other (with ethanol as lubricant between PDMS and glass nozzles).

### 2.4 Device mounting and flow monitoring

The experimental setup with the assembled microfluidic devices were illustrated in Fig. S3.† The core channel was placed at the vertical direction, to facilitate the gravity-driven collection of as-spun fibers, as well as microscopic imaging. The fully transparent PDMS and glass components allow direct monitoring of flow behaviors within the microfluidic chip. To visualize the fluid flows, molecular dyes of different colors were used for mixing with polymer solutions. Digital micrographs and movies were taken by a Leica DMS300 optical microscopy.

### 2.5 General fiber spinning procedures

Before initiating alginate (Alg) hydrogel fiber spinning, stable co-axial laminar flows were firstly established with DI water as sheath layer. Briefly, the DI water sheath flow was firstly initiated at the given flow rate (*Q*_sheath_), Alg flow (*e.g.* core flow in single-module chip, or shell flow in dual-module chip, or 2nd shell flow in triple-module chip) was then started at the given flow rate. The further inner layers were initiated consecutively (*e.g.* core flow in dual-module chip, or 1st shell flow and core flow in triple-module chip). All the flows were controlled by high-accuracy and pulseless syringe pumps (Nemesys® system from CETONI GmbH, Germany). The established laminar flow at each step was visualized by 30 second microscopic video, confirming the stable layered flow configurations. Subsequently, by switching the DI water flow to CaCl_2_ (570 mM) aqueous solution, the Alg polymer solution (2 wt% in DI water) will be solidified by Alg–Ca^2+^ crosslinking-induced gelation. A Y-junction was installed for the abovementioned switch between DI water and CaCl_2_ flows from the side inlet, which facilitated the smooth starting and ending of fiber spinning, thus lowering the risk of device clotting. Furthermore, in instances of clotting during fiber spinning, the CaCl_2_ flow was switched back to DI water immediately upon the occurrence of the clotting event. This allowed for quick cleaning of the device and prompt resumption of fiber spinning. No leakage was observed unless clotting was allowed to persist without being cleaned in this manner.

For the spinning of hollow Alg hydrogel fibers using a dual-module device, a PEG (Mw. 900 kD, 2 wt%) solution was used as core flow, which acted as a non-solidifying temporal template and was removed after spinning to form the hollow core. For the spinning of multimaterial fibers with a tri-module device, a FTIC–chitosan-containing Alg solution (FITC–chitosan 0.1 wt%, Alg 2 wt%) was used for the 1st shell flow, in combination with a gelatin solution (30 wt%) for core flow and a mixture solution of acrylamide monomers (40 wt%), *N*,*N*′-methylenebis(acrylamide) crosslinker (0.06 wt%) and Irgacure 2959 photoinitiator (0.05 wt%) for the 2nd shell flow, and CaCl_2_ solution (0.7 wt%) as sheath flow.

### 2.6 Synthesis of FITC–chitosan

Chitosan 85/100 was stirred for 2 hours in 1% acetic acid solution and subsequently neutralized to pH 6 by the addition of NaOH (aq., 1 M). Fluorescein isothiocyanate (FITC) was added to the chitosan solution (final concentration 4.4 mM) and the reaction was stirred overnight in the dark at room temperature. The labelled chitosan (FITC–chitosan) was precipitated by adding NaOH (aq. 1 M) and filtered. The residue was washed with NaOH (aq., 5 mM) and then resuspended in DI water. The final FITC–chitosan was stored at 4 °C as a stock solution with a concentration of 1% w/v (51 μM of FITC, conjugation yield 42%).

### 2.7 Leakage test of pH-sensing fibers

After preparation, three specimens of the fibers (2 cm each) were cut and soaked in 1 mL of PBS (pH 7.4). The fluorescent signal (emission at *λ* = 517 nm) of the solution was measured weekly (excitation *λ* = 498 nm) using the fluorescent spectrophotometer (Cary Eclipse, Agilent, Santa Clara, CA, US).

### 2.8 pH-Responsiveness of the fiber

The prepared fiber (2 cm in length) was placed in a 96-well plate and 100 μL of PBS solution at defined pH was added. After 1 hour of incubation, the fluorescent signal (*λ* = 517 nm) was measured (with excitation *λ* = 498 nm, Cary Eclipse, Agilent, Santa Clara, CA, US), the solution was removed and replaced with 100 μL of PBS at different pH. The experiment was accomplished in triplicate and each fiber was tested with the PBS solution at pH 5, 6, 7, and 8.

## 3. Results and discussion

### 3.1 Microfluidic device design and fabrication

With a Lego® plate as a base, numerous different modules can be assembled and aligned together to form an integrated design (Fig. 2). PDMS blocks with a built-in core channel and Lego® features on the bottom were developed with a molding procedure according to the method described in Section 2.1 and 2.2, as well as in the ESI† (Fig. S1 and S2). The negative Lego® stud feature on the bottom of the PDMS blocks ensured the facile assembly of multiple PDMS-nozzle modules on a Lego® plate. The accurate alignment of the glass nozzles from different modules was guided by the multi-point Lego® stud features. On the one hand, this allowed the concentric configuration of the nozzles and capillaries, without the use of special guiding glass tubes (*e.g.*, rectangular tube), sealing connectors, or chemical adhesives.^12^ On the other hand, the glass nozzles and capillary were accessible when the device was disassembled into individual modules. This ensured easy cleaning, de-clotting, and adjusting the device configuration, as well as replacing individual parts of the system if necessary.

### 3.2 Spinning with a single-module device

As a proof-of-concept, the Alg–Ca^2+^ hydrogel was chosen for hydrodynamic spinning with our PnP microfluidic device, as Alg–Ca^2+^ hydrogels have been widely used in many biomedical studies such as tissue engineering, biosensing, and drug delivery, due to their excellent biocompatibility and straightforward preparation.^34,35^ Particularly, it is one of the most commonly used materials for the fabrication of hydrogel fibers,^1^ which have been demonstrated for encapsulating cells,^12^ nanomaterials,^36^ and stimuli-responsive polymers.^6,37^ Unlike polyethylene glycol diacrylate (PEGDA) and gelatin-methacryloyl (GelMA) that need extra gelating procedures triggered by photo-initiated polymer crosslinking,^38,39^ Alg gelates instantaneously upon mixing with Ca^2+^ due to the rapid ionic crosslinking of Alg polymers. Therefore, combined with its lower cost than that of PEGDA and GelMA, Alg has been a rational choice for many pioneering studies on hydrogel fibers.

**Fig. 3:**
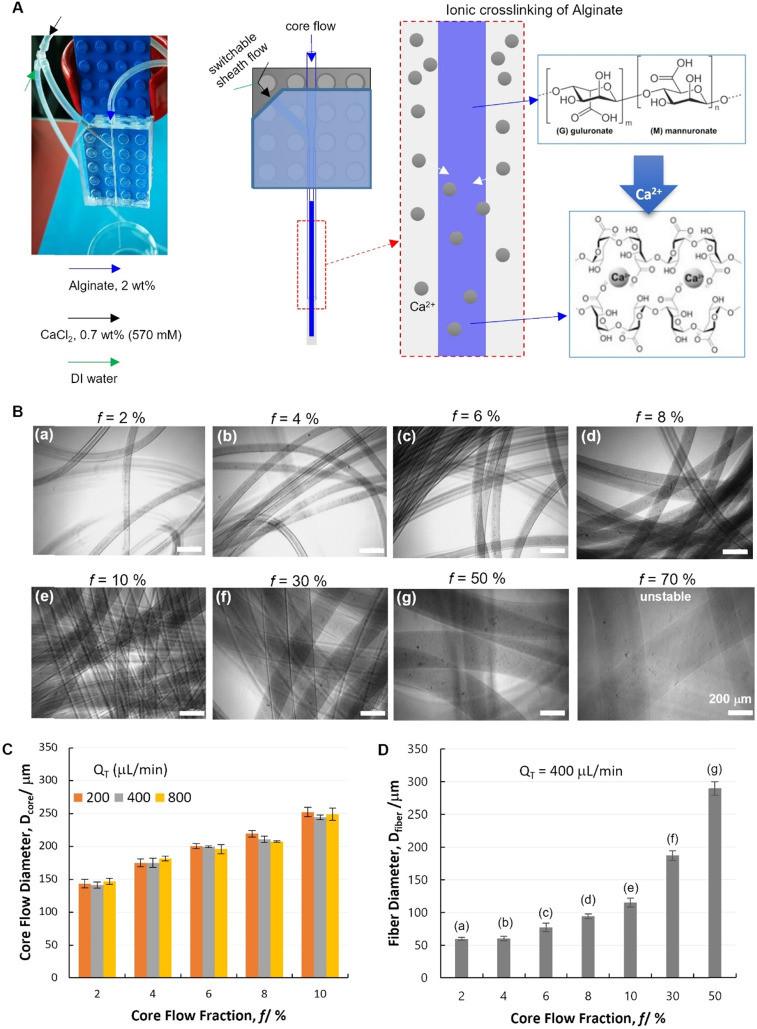
Spinning of alginate hydrogel fibers with a single-module device. (A) A digital micrograph and a schematic illustration of the single-module microfluidic device configuration for spinning ionically crosslinked alginate hydrogel fibers. (B) Digital micrographs of hydrogel fibers spun at different flow conditions (with *Q*_T_ = *Q*_core_ + *Q*_shell_ fixed at 400 μL min^−1^, and varying core flow fraction *f* = *Q*_core_/*Q*_T_). (C) Flow behaviors in the single-module device characterized by the core flow diameter (*D*_core_) in response to the flow rate variation. (D) Diameter of hydrogel fibers (*D*_fiber_) spun at different flow conditions. In panel B and D, different spinning conditions were labelled with (a)–(g).

As a first step, a single-module device was used for spinning of Alg hydrogel fibers. A core flow of Alg solution (2 wt%) was introduced from the glass nozzle (blue), which was solidified by Alg–Ca^2+^ ionic crosslinking in the outlet capillary, where the Alg/Ca^2+^ core–shell laminar flow was established and Ca^2+^ diffusion to the core flow occurred (Fig. 3A). It is noteworthy that, the fluid flows were established by using high-accuracy and pulse-free CETONI pumps with programmable controls, which enables the on-demand switching between different fluids. Herein, a Y-junction was installed for a switchable shell flow from the side inlet, which facilitated the smooth starting and ending of fiber spinning, and lowered the risk of device clotting. Specifically, the core–shell laminar flow was firstly established with DI water as shell flow (Fig. 3A, green arrow). Since no gelation can be triggered at this stage, the uncertain flow behaviors at the onset of experiments were avoided to cause device clotting. At the second stage, only after the stable laminar flow was established between Alg solution and DI water, the shell flow was switched to Ca^2+^ solution (Fig. 3A, black arrow) to form hydrogel fibers. At last, to stop fiber spinning, the shell flow was switched back to DI water until no fiber but only the solution was coming out from the outlet capillary. As such, unwanted gel-state residual materials was avoided in the device.

This single-module device was demonstrated with its ability to control the core flow diameter (*D*_core_), which is a key prerequisite for the spinning of diameter-defined hydrogel fibers (*D*_fiber_). It has been well documented that the core flow dimensions are controlled by both core flow and shell flow rates (*Q*_core_ and *Q*_shell_, respectively) with such co-axial hydrodynamic focusing flow configurations.^11,40^ It has been also revealed in our previous study by computational simulation that, *D*_core_ increases with increasing *Q*_core_, or with decreasing *Q*_shell_.^41^ Herein, such a phenomenon was confirmed experimentally by quantifying *D*_core_ under different flow conditions. Specifically, the core flow fraction was defined as *f* = *Q*_core_/*Q*_T_, where *Q*_T_ indicates the total fluid flow rate (*Q*_T_ = *Q*_core_ + *Q*_shell_). It was found that *D*_core_ increases significantly with increasing *f* (Fig. 3C, and Table S1,† 2-factor with replication ANOVA, *P*-value 6.6 × 10^−24^ < 0.05). The variation of *Q*_T_ is found not relevant to *D*_core_ (*P*-value 0.30 > 0.05). At each fixed *f*, the core–shell flow behavior remained unaffected by *Q*_T_ at 200, 400 and 800 μL min^−1^. Particularly, it is noteworthy that, this flow behavior is of great interest for hydrodynamic spinning of polymer fibers, since it indicates that, fiber dimensions (defined by *f*) could potentially be decoupled with other fiber properties related to the production speed (defined by *Q*_T_). Such an independent control of different properties is important for practical applications of fiber materials. According to the abovementioned flow dimeter control, a range of Alg hydrogel fibers were spun with different *D*_fiber_ by switching the shell flow to CaCl_2_ solution (Fig. 3B and D).

### 3.3 Spinning with a dual-module device

**Fig. 4:**
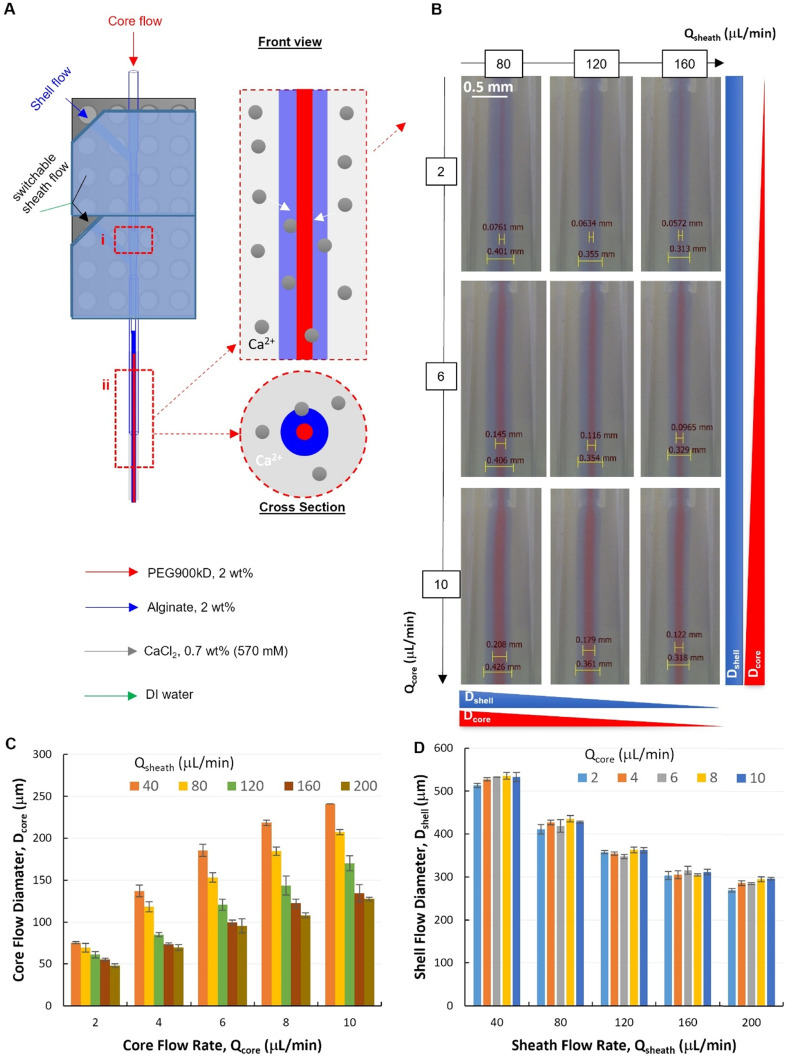
A dual-module device for hollow fiber spinning. (A) A schematic illustration of the dual-module microfluidic device configuration with two hydrodynamic focusing positions labelled with (i) and (ii). (B) Micrographs of tri-layered flows (at focusing position ii) with 2 wt% PEG900kD in DI water as core flow, 2 wt% Alg in DI water as shell flow and deionized (DI) water as sheath flow. (C and D) Quantitative analysis of core and shell flow diameter at 2nd focusing, respectively (*Q*_T_ = *Q*_core_ + *Q*_shell_ = 40 μL min^−1^).

Two modules were assembled to verify our modular design of the PnP device, which is of great interest for complex flow configurations and fiber micro-structures (Fig. 4). The dual-module device (Fig. S4†) showed high capability to establish and control with high quality the formation of a stable core–shell–sheath laminar flow (ESI,† Movies S1 and S2). To investigate the capacity of such a modular microfluidic platform in manipulating the fluid flow, a tri-layer in-channel core–shell–sheath flow configuration was established by using PEG (Mw. 900 kD, 2 wt%, red), Alg (2 wt%, blue), and DI water as the core, shell, and sheath fluids, respectively.

The flow behaviors at the second focusing position was investigated, where the Alg hydrogel layer is supposed to be formed *via* Alg–Ca^2+^ crosslinking (Fig. 4A). It was demonstrated that, at the second focusing position (Fig. 4A-ii), the quality of the modular microfluidic device allowed for establishing the core–shell–sheath tri-layered flow structure, where the dimensions of the polymer flows can be manipulated by flow rates (Fig. 4B). Interestingly, quantitative analysis (Fig. 4C and D) revealed that, at the second focusing position, in addition to the main effect from *Q*_core_ (with *Q*_T_ = 40 μL min^−1^), *Q*_sheath_ showed also influence on *D*_core_. At given sheath flow rates (*Q*_sheath_ ranging from 40 to 200 μL min^−1^), core flow diameter increases significantly with increasing *Q*_core_ (2-factor with replication ANOVA, *P*-value 1.10 × 10^−42^ < 0.05, Table S2†). Meanwhile, at given *Q*_core_, *D*_core_ decreased with increasing *Q*_sheath_, which appears to be also a significant effect (*P*-value 5.18 × 10^−37^ < 0.05). In addition, the interaction between the effects of *Q*_core_ and *Q*_sheath_ on *D*_core_ was found significant (*P*-value 3.97 × 10^−17^ < 0.05). On the other hand, *D*_shell_ depends mainly on *Q*_sheath_, while also affected by changing *Q*_core_ (Fig. 4D). It was found that both effects of *Q*_core_ and *Q*_sheath_ on *D*_core_ were significant, but they had no significant interactions with each other (2-factor with replication ANOVA, Table S3†). This indicates that the core–shell polymer flows can be well configured into different dimensions by tuning independently *Q*_core_ and *Q*_sheath_, which is important for controlling fiber structures spun with such flow configurations.

It is noteworthy that, at the first focusing position (Fig. 4A-i), the core–shell flow configuration was not affected by the sheath flow introduced from the second module, but by the core and shell flow rates (*Q*_core_ and *Q*_shell_). Specifically, at an exemplified *Q*_T_ (*Q*_T_ = *Q*_core_ + *Q*_shell_ = 40 μL min^−1^) and increasing *Q*_sheath_ (ranging from 40–200 μL min^−1^), the core flow diameter (*D*_core_) remained unchanged at each fixed *Q*_core_. Consistent with the flow behaviors from the single-module device, *D*_core_ increased with the increasing core flow rate (*Q*_core_ ranging from 2–10 μL min^−1^), as shown in the representative flow images and the corresponding quantitative analysis (Fig. S5 and S6†).

**Fig. 5:**
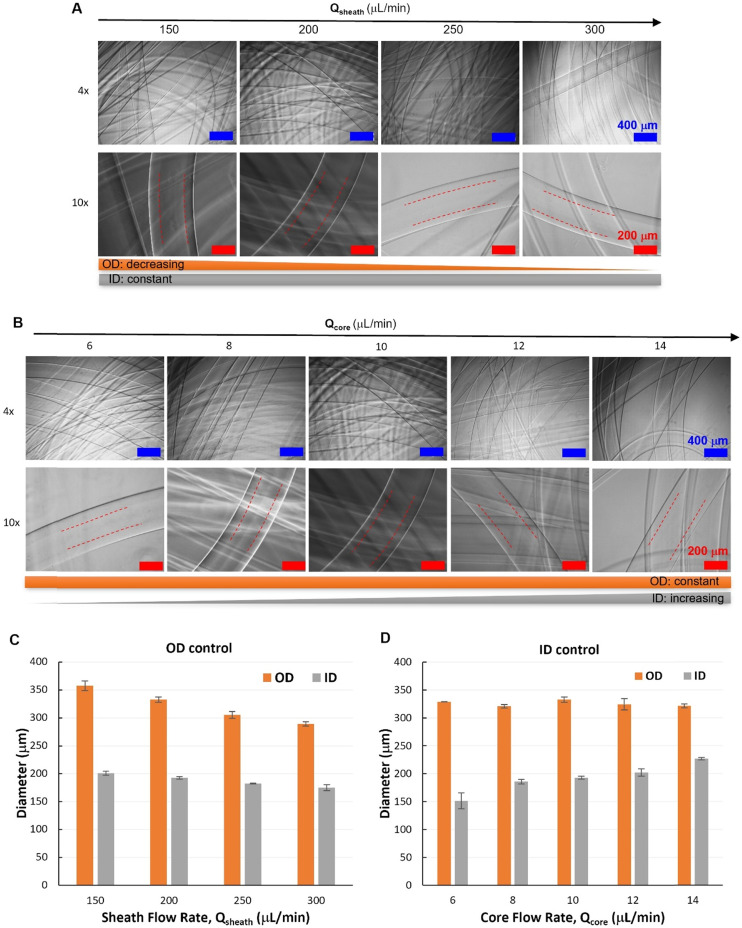
Hollow hydrogel fibers with different outer diameters (OD) or inner diameters (ID). (A) Micrographs of hollow fibers with OD controlled by *Q*_sheath_ at constant *Q*_core_ (10 μL min^−1^) and *Q*_shell_ (30 μL min^−1^). (B) Micrographs of hollow fibers with ID controlled by *Q*_core_ at constant *Q*_shell_ (30 μL min^−1^) and *Q*_sheath_ (200 μL min^−1^). (C and D) Quantitative analysis of hollow fibers with OD controlled by *Q*_sheath_ (A) or ID controlled by *Q*_core_.

After the core–shell–sheath flow configuration was established, hollow Alg hydrogel fibers were produced by changing the switchable sheath flow from DI water to CaCl_2_ solution, whereby the diffusion of Ca^2+^ from the sheath flow to the shell flow (Alg solution) induced rapid crosslinking of Alg polymers. Similar to the spinning of filled fibers, the unwanted device clotting was avoided by the controlled switching of sheath flow from water to CaCl_2_ solution. According to the flow behaviors, hollow hydrogel fibers with varied outer diameters (OD) but similar inner diameters (ID) were spun by changing only *Q*_sheath_ (150–300 μL min^−1^), while maintaining constant *Q*_core_ (10 μL min^−1^) and *Q*_shell_ (30 μL min^−1^). Representative optical micrographs showed the hollow nature of such fibers with smooth outer and inner surfaces (Fig. 5A and B), which could be visualized by protein (FITC-BSA) encapsulation (Fig. S7†). Quantitative image analysis (Fig. 5C and D) revealed that hydrogel fibers spun under such conditions had similar ID with a slight decrease at higher *Q*_sheath_ (250 and 300 μL min^−1^), while OD decreased from 357 ± 8 μm (*Q*_sheath_ = 150 μL min^−1^) to 289 ± 4 μm (*Q*_sheath_ = 300 μL min^−1^). Alternatively, fibers with similar OD can be spun with varied ID controlled by *Q*_core_. With fixed *Q*_shell_ (30 μL min^−1^) and *Q*_sheath_ (200 μL min^−1^), fiber ID increased from 151 ± 14 μm (*Q*_core_ = 6 μL min^−1^) to 219 ± 6 μm (*Q*_core_ = 14 μL min^−1^).

### 3.4 Spinning with a triple-module device

**Fig. 6:**
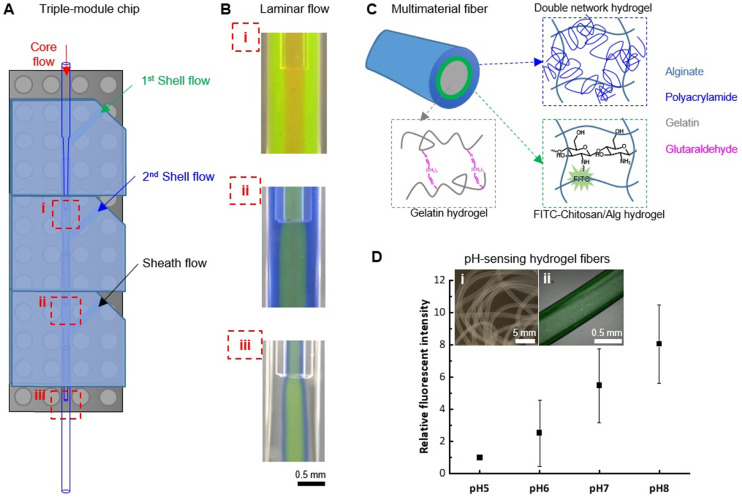
Spinning of multimaterial fibers with a tri-module microfluidic device. (A) A schematic illustration of the tri-module microfluidic device configuration with three hydrodynamic focusing positions labelled with (i)–(iii). (B) Stable co-axial flows established at all three hydrodynamic focusing positions, visualized by adding to 2 wt% Alg solution with rhodamine B (red), fluorescein (green), and aniline blue (blue) to the core, 1st shell, and 2nd shell flow, respectively. (C) Schematic illustration of the structure of the tri-layered hydrogel fibers. (D) pH-Responsiveness of the hydrogel fibers (insert i: a micrograph of the tri-layered fibers; ii: a confocal laser scanning microscopy image of the pH-sensing fiber in PBS, pH 7.4). Values are reported as average (*n* = 3) ± standard deviation.

As a modular system, a characteristic feature is the facile assembly of additional modules for fast development of upgraded devices. As a proof-of-concept, a triple-module device was demonstrated for manipulating more complex co-axial flows of polymer solutions and spinning of multimaterial hydrogel fibers (Fig. S8†). Fabricated by simply assembling three modules on a Lego® base, the triple-module device allowed the introduction of a 2nd shell flow, in addition to the core, 1st shell, and sheath flows that can be manipulated by a dual-module device. Therefore, three hydrodynamic focusing positions can be used for configuring the co-axial polymer flow structure (Fig. 6A). To validate the quality of such a triple-module device, the polymer flows (2 wt% Alg solution) were visualized by adding rhodamine B (red), fluorescein (green), and aniline blue (blue) to the core, 1st shell, and 2nd shell flow, respectively. The colorless flow can be switched between DI water and CaCl_2_ solutions, so that the start/stop of fiber spinning can be controlled and undesired channel clotting can be avoided. The stable co-axial flows established at all three hydrodynamic focusing positions (Fig. 6B) confirmed that the quality of the nozzle co-axial alignment was provided by the PnP device assembly strategy. In particular, the 4-layered flow structure (*i.e.* the red core flow, the green 1st shell flow, the blue 2nd shell flow, and the colorless sheath flow) established at the focusing position-iii was crucial for dictating the morphology of hydrogel fibers, therefore establishing and maintaining the co-axial flow stability is of key importance, which is demonstrated by a 30 second video recording the 4-layered flow structure at this position (ESI,† Movie S3).

As an example of the application of such a triple-module device, multimaterial tri-layered hydrogel fibers with pH-sensing functionality were fabricated (Fig. 6C). For this purpose, fluorescein isothiocyanate-conjugated chitosan (FITC–chitosan) was synthesized as pH-sensing component.^42^ The stable entrapment of the pH-sensitive component was ensured by the electrostatic interactions between chitosan (positively charged) and alginate (negatively charged). To embed it between a cell-compatible hydrogel core, which could potentially host living cells, and a mechanically robust hydrogel sheath, a FTIC–chitosan-containing Alg solution was used for the 1st shell flow, in combination with a gelatin solution for core flow and a mixture solution of acrylamide monomers, *N*,*N*′-methylenebis(acrylamide) crosslinker and Irgacure 2959 photoinitiator for the 2nd shell flow (see Section 2.5). With CaCl_2_ solution as sheath flow, hydrogel fibers were formed with Alg–Ca^2+^ crosslinks. The gelatin core was subsequently crosslinked in the aqueous collection bath containing glutaraldehyde. The *in situ* formation of the alginate–polyacrylamide double-network hydrogel sheath layer was induced by UV (*λ* = 365 nm) irradiation. In between the gelatin core and the double-network sheath, the pH-sensing component FITC–chitosan was stabilized in Alg–Ca^2+^ hydrogel layer (Fig. 6D, inserts). Up to 21 days of incubation in PBS (pH 7.4), no substantial leakage of FTIC–chitosan was observed from the fibers confirming the stable incorporation of the functional component (Fig. S9†). The pH-responsiveness of the so-obtained fiber was tested by treating it with PBS solutions at biologically relevant pHs, *i.e.* pH 5, 6, 7, and 8, representing the pH range of wound exudate from low (pH ∼5) for healthy or healing wound to alkaline (pH ∼8) for chronic, infected wounds.^43,44^ Within this range, the fluorescent signal of the fiber increased with the alkalinity of the solution. The relative fluorescent intensity, calculated using the intensity measured at pH 5 as the relative value (*I*_pH*n*_/*I*_pH5_), showed an 8-fold increment of the signal when treated with pH 8 (Fig. 6D), thus suggesting the potential application in pH-sensing for biomedical applications. We have recently demonstrated that fluorescein-based sensors are well suited for the monitoring of pH in wound exudates and can be incorporated into lab-on-a-fiber wearable patches.^43^ Our multimaterial fiber could also be used in such a setting, with the additional advantage that no further post-processing functionalization step is required.

## 4. Conclusion

The design and fabrication of a modular microfluidic platform was described in this report. Its application in hydrodynamic spinning of hydrogel fibers was demonstrated. Without the need for special instruments such as high-resolution 3D printers and clean room facilities, such platforms can be fabricated in laboratories with only basic settings (*i.e.* ovens for PDMS elastomer curing). While other components (*i.e.* glass nozzles and capillaries, Lego® parts) are commercially available, the design can be easily adapted by a broad range of would-be users. The “plug-and-play” design allows rapid device fabrication (*via* modular assembly) and easy device reparation (*via* disassembly and re-assembly). With the unique modular feature and precise flow manipulating capacity, the microfluidic platform can be adapted for different biomedical applications, and it is particularly promising for biomedical researchers as an easy and cost-effective method for their development of microfiber biomaterials and tissue engineering scaffolds.

## Data availability

The authors confirm that the data supporting the findings of this study are available within the article and as its ESI.†

## Author contributions

Kongchang Wei: conceptualization, data curation, formal analysis, investigation, methodology, validation, visualization, writing – original draft, writing – review & editing, Wuchao Wang: data curation, formal analysis, investigation, methodology, validation, visualization, writing – review & editing, Giorgia Giovannini: data curation, formal analysis, investigation, methodology, validation, visualization, writing – review & editing, Khushdeep Sharma: investigation, methodology, validation, writing – review & editing, René M. Rossi: conceptualization, investigation, project administration, resources, supervision, validation, writing – review & editing, Luciano F. Boesel: conceptualization, formal analysis, funding acquisition, project administration, resources, supervision, validation, writing – review & editing.

## Conflicts of interest

On behalf of all authors, the corresponding author states that there is no conflict of interest.

## Supplementary Material

LC-025-D4LC00783B-s001

LC-025-D4LC00783B-s002

LC-025-D4LC00783B-s003

LC-025-D4LC00783B-s004
