# Supplementary material for: A plug-and-play microfluidic device for hydrogel fiber spinning
Source: Lab Chip. 2025 Feb 6;25(6):1575–85. doi: 10.1039/d4lc00783b (PMC11815318; doi:10.1039/d4lc00783b)
Supplement: LC-025-D4LC00783B-s004 [file LC-025-D4LC00783B-s004.pdf]

Supplementary Information (SI) for Lab on a Chip.
This journal is © The Royal Society of Chemistry 2025

Supporting Information

A Plug-and-Play Microfluidic Device for Hydrogel Fiber Spinning

Kongchang Wei,[1,2] Wuchao Wang,[1] Giorgia Giovannini,[1] Khushdeep Sharma,[1] René M. Rossi,[1,*] Luciano F. Boesel[1,*]

[1] Empa, Swiss Federal Laboratories for Materials Science and Technology, Laboratory for Biomimetic Membranes and Textiles, Lerchenfeldstrasse 5, 9014 St. Gallen, Switzerland

[2] Empa, Swiss Federal Laboratories for Materials Science and Technology, Laboratory for Biointerfaces, Lerchenfeldstrasse 5, 9014 St. Gallen, Switzerland

Corresponding authors:

*L. F. Boesel: luciano.boesel@empa.ch

*R. Rossi: rene.rossi@empa.ch

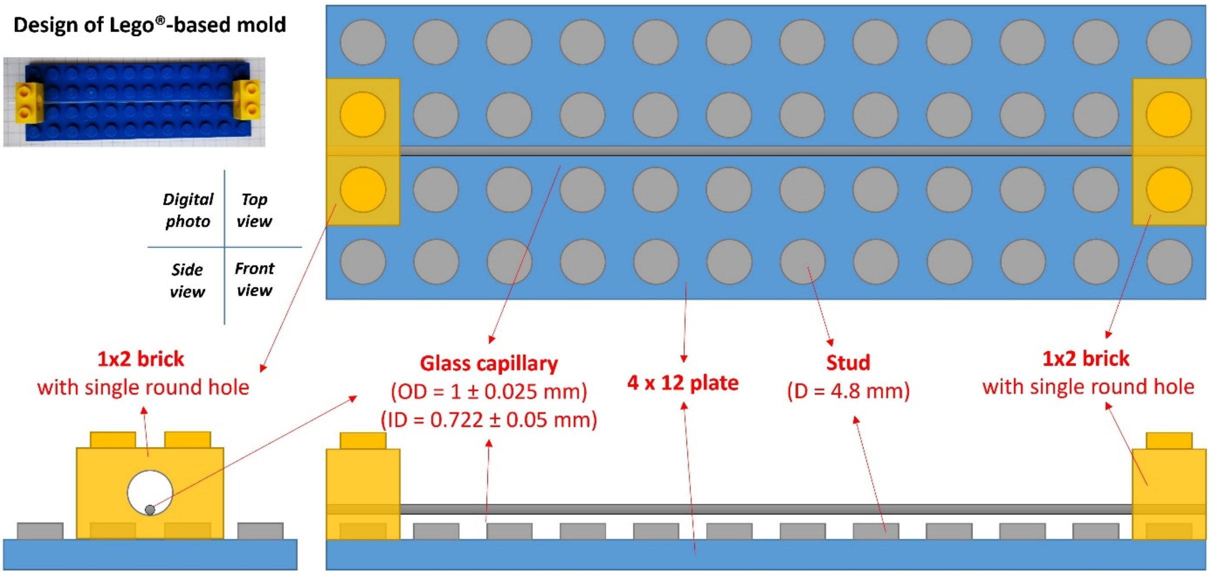

Figure S1. The design and assembly of the Lego®-based mold for the fabrication of PDMS microfluidic blocks.

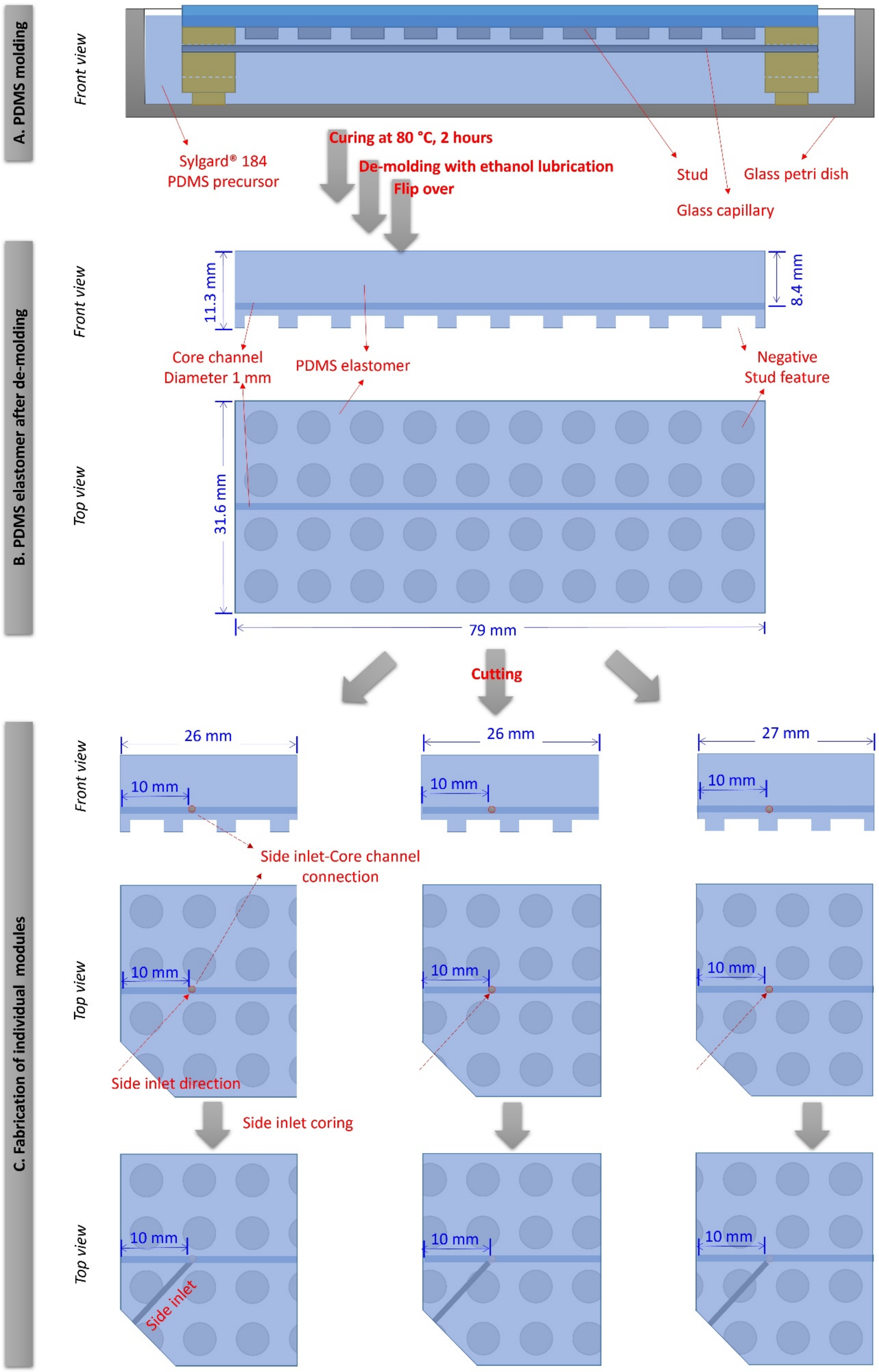

Figure S2. Molding (A), de-molding (B), cutting of the PDMS microfluidic block with a core channel and negative Lego® stud features, followed by the coring out of side inlets with an 18G cone needle (C).

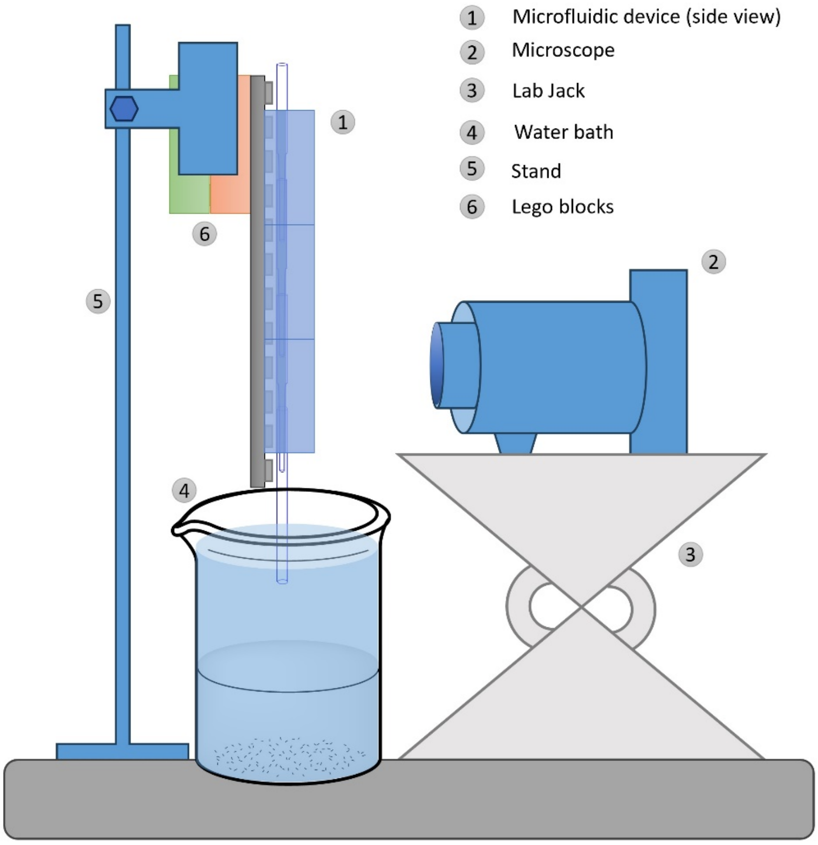

Figure S3. Experimental setup for microfluidic wet spinning of hydrogel fibers.

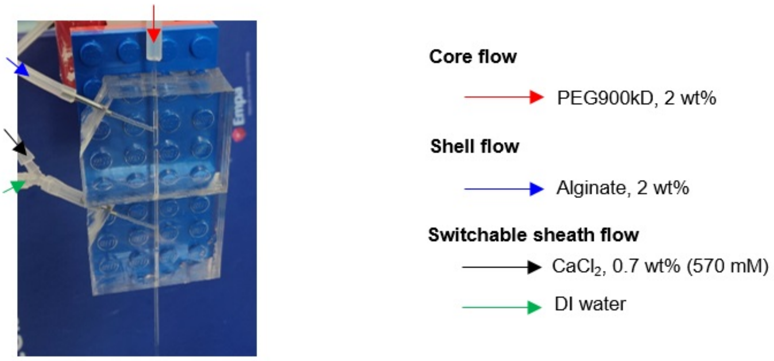

Figure S4. A digital photo of a dual-module device with the flow configuration used for spinning hollow alginate hydrogel fibers.

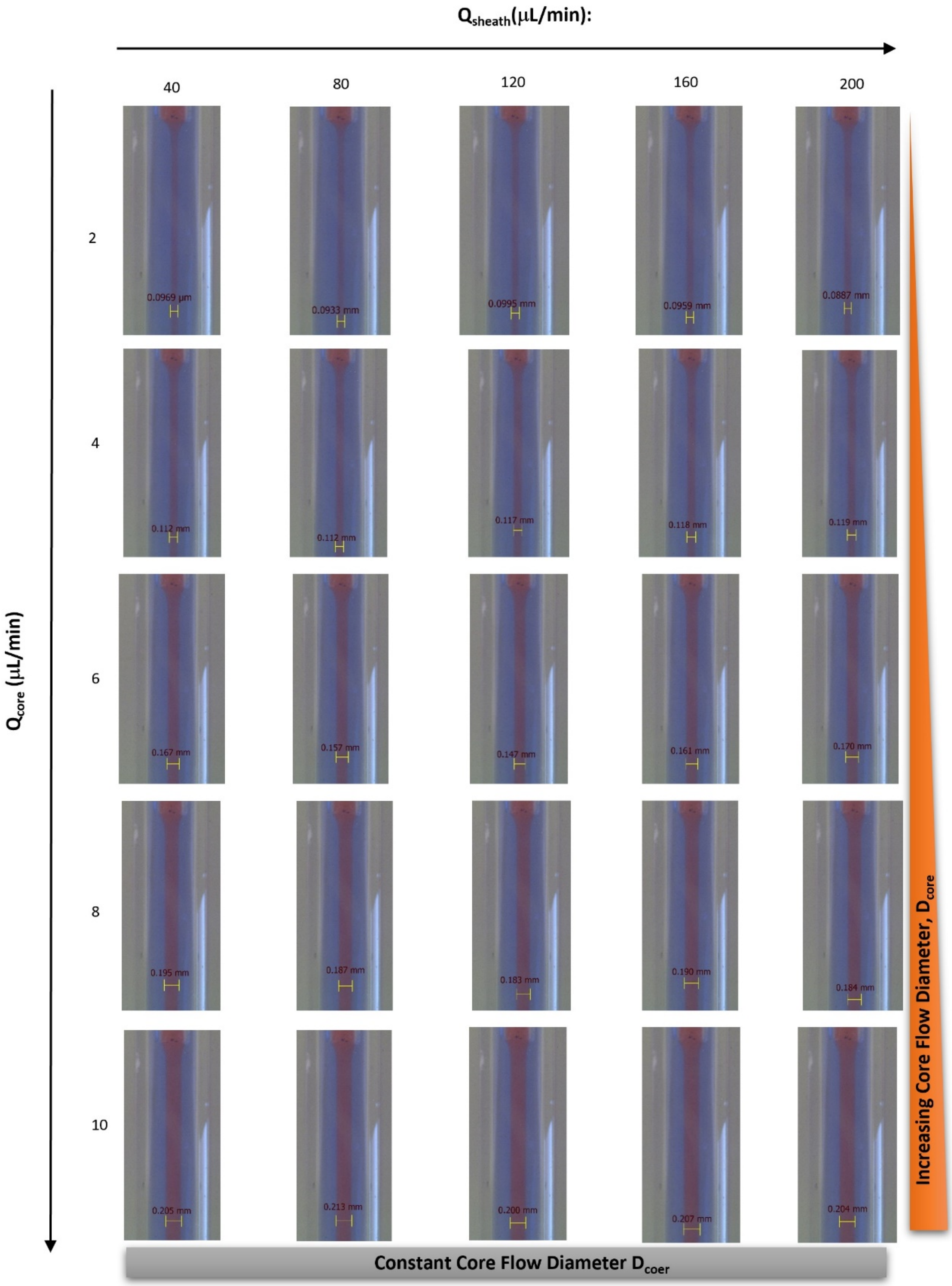

Figure S5. First focusing (indicated in Fig. 4A-i) core-shell flow established by the dual-module device, when $Q_T = Q_{core} + Q_{shell}$ was fixed at 40 μL/min under different Q_{sheath}.

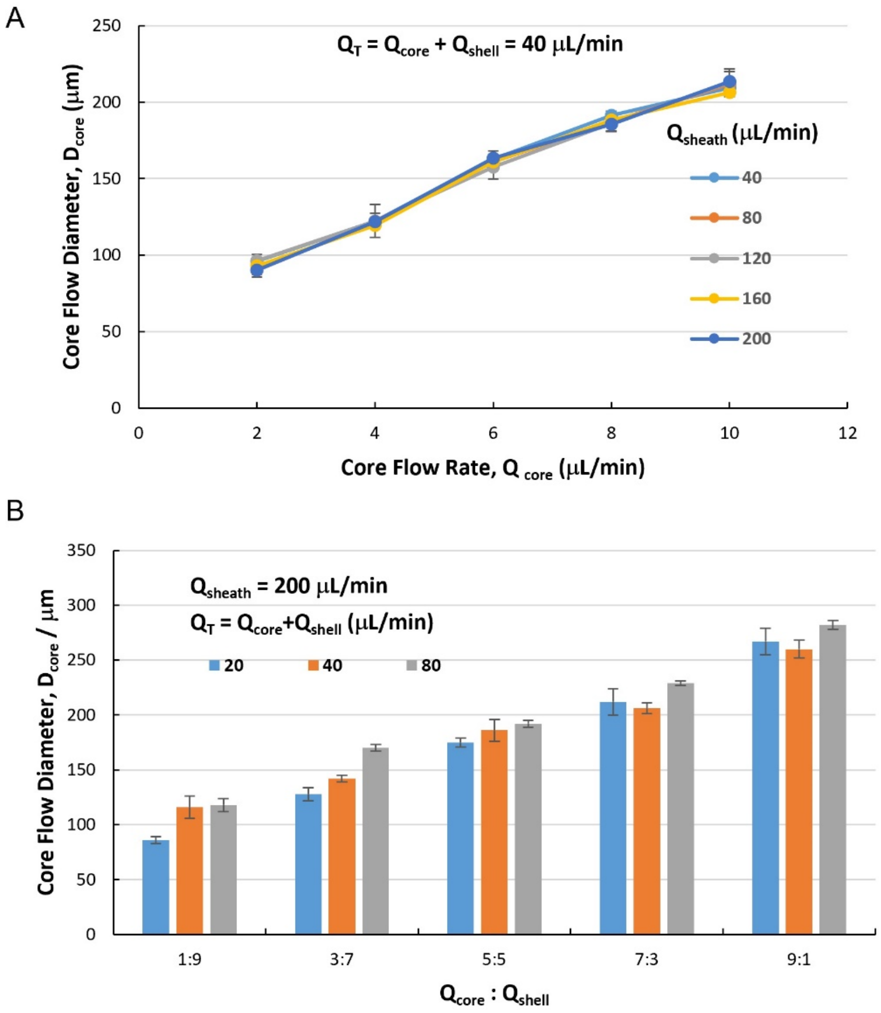

Figure S6. (A) Quantification of the diameter of core flows (from Figure S4, when $Q_T = Q_{core} + Q_{shell}$ was fixed at 40 μL/min) under different Q_{sheath}, showing that sheath flow rate has negligible influence on core flow diameter at 1st focusing. (B) Quantification of the diameter of core flows (from Figure S3-A, when Q_{sheath} was fixed at 200 μL/min, and Q_T varied as 20, 40 or 80 μL/min), showing that Q_T has negligible influence on core flow diameter at 1st focusing.

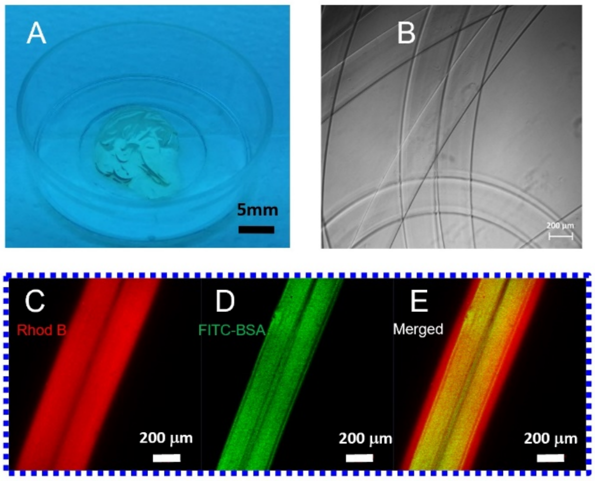

Figure S7. Hollow nature of the hydrogel fibers (spun with double-module device) characterized by protein (FITC-BSA) encapsulation.

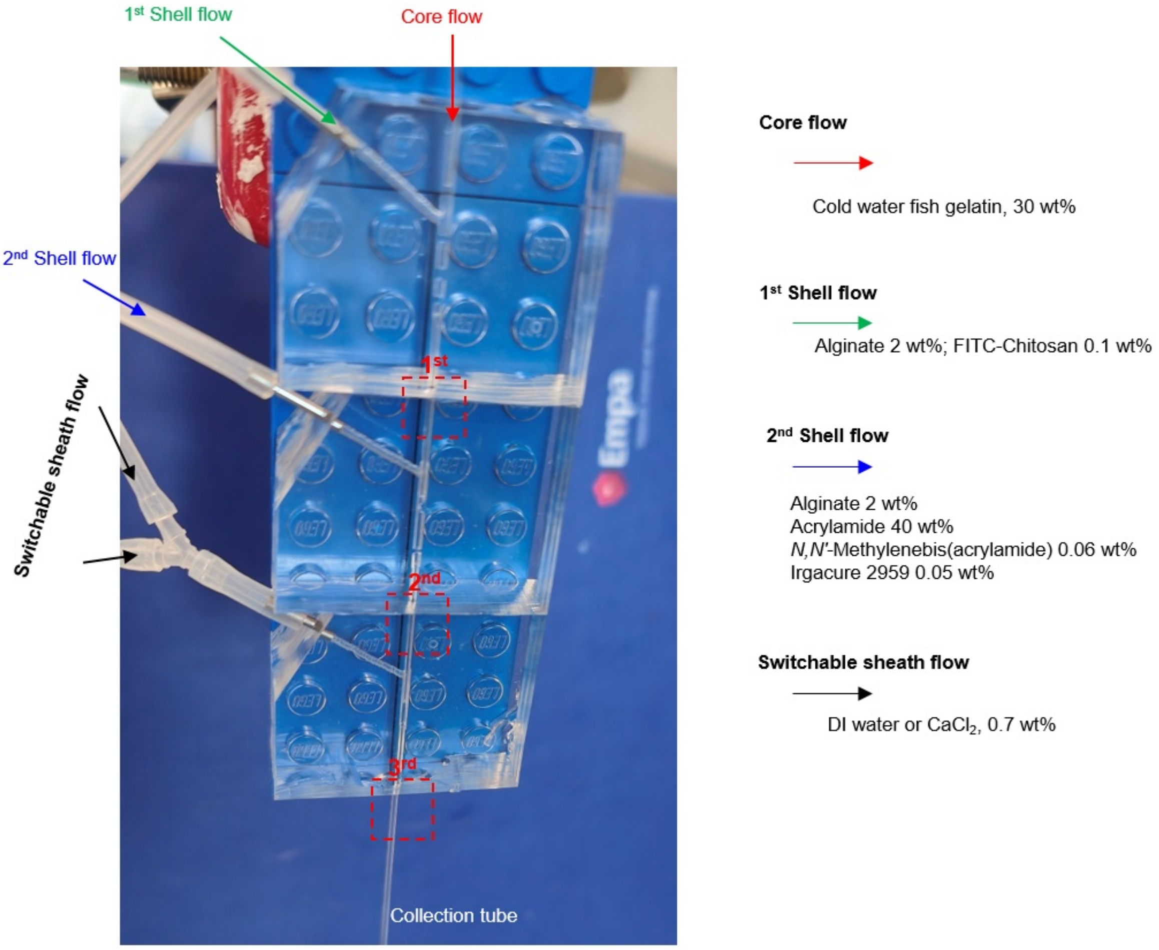

Figure S8. A digital photo of the tri-module device and the flow configuration for spinning pH-sensing multimaterial hydrogel fibers.

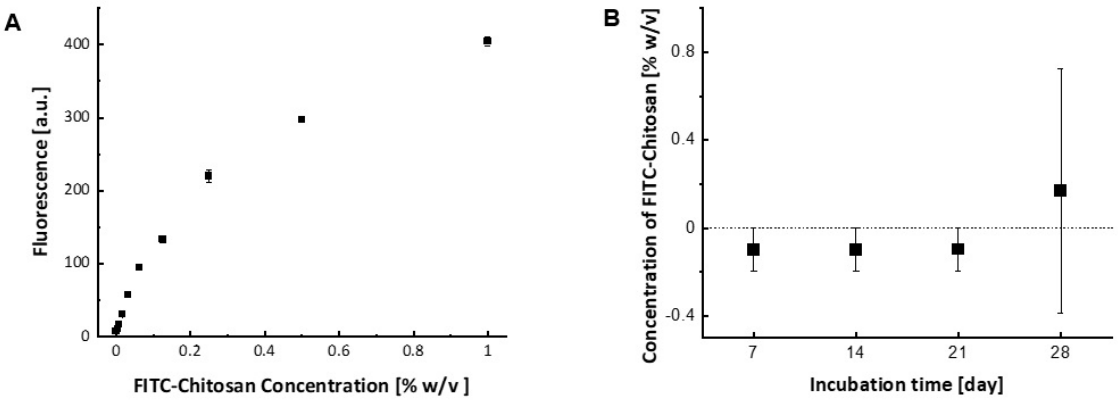

Figure S9. (A) Standard curve of FITC-Chitosan fluorescence according to its concentration at pH 7.5 PBS. (B) Linkage of FITC-Chitosan from fibers to at pH 7.5 in PBS.

Table S1. For affecting core flow diameter (D_{core}) from single-module chip (Fig. 3A), the total fluid flow rate ($Q_T = Q_{core} + Q_{shell}$) is found not statistically significant (*P*-value 0.29 > 0.05), while the core flow fraction *f* ($f = Q_{core}/Q_T$) is found statistically significant (*P*-value 6.63E-24 < 0.05).

ANOVA: Two-Factor With Replication

Source of Variation	*SS*	*df*	*MS*	*F*	***P-value***	*F crit*
Sample (Q_T)	112.52	2	56.26	1.26	**0.30**	3.32
Columns (f)	55077.84	4	13769.46	308.65	**6.6E-24**	2.69
Interaction	367.17	8	45.90	1.03	**0.4367**	2.27
Within	1338.34	30	44.61			
Total	56895.87	44				

Table S2. For affecting core flow diameter (D_{core}) from dual-module chip (Fig. 5B), both the core flow rate (Q_{core}) and the sheath flow rate (Q_{sheath}) are found statistically significant effects on core flow diameter (D_{core}), with *P*-values < 0.05. A significant interaction between the effects of Q_{core} and Q_{sheath} on D_{core} is indicated by *P*-value of 3.97E-17 < 0.05.

ANOVA: Two-Factor With Replication

Source of Variation	*SS*	*df*	*MS*	*F*	***P-value***	*F crit*
Sample (Q_{core})	120993.2	4	30248.3	666.07	**1.10E-42**	2.56
Columns (Q_{sheath})	70785.02	4	17696.25	389.67	**5.18E-37**	2.56
Interaction	15829.03	16	989.31	21.78	**3.97E-17**	1.85
Within	2270.667	50	45.41			
Total	209877.9	74				

Table S3. For affecting shell flow diameter (D_{shell}) from dual-module chip (Fig. 5C), both the core flow rate (Q_{core}) and the sheath flow rate (Q_{sheath}) are found statistically significant with *P*-values < 0.05. However, the interaction between these two factors is not significant, with a *P*-value of 0.1 > 0.05

ANOVA: Two-Factor With Replication

Source of Variation	*SS*	*df*	*MS*	*F*	***P-value***	*F crit*
Sample (Q_{sheath})	574552.10	4	143638	2055.50	**8.84E-55**	2.56
Columns (Q_{core})	2467.73	4	616.93	8.83	**1.80E-05**	2.56
Interaction	1810.13	16	113.13	1.62	**0.10**	1.85
Within	3494	50	69.88			
Total	582324	74				

Movie S1: Manipulation of core-shell flow with a dual-module chip, where the core flow diameter was maintained constant, while the shell flow diameter was changed independently.

Movie S2: Manipulation of core-shell flow with a dual-module chip, where the shell flow diameter was maintained constant, while the core flow diameter was changed independently.

Movie S3: Stable 4-layer flow established with the triple-module microfluidic chip.

Reference

1 A. M. Christensen, D. A. Chang-Yen and B. K. Gale, *J. Micromechanics Microengineering*, 2005, **15**, 928–934.
